# A Novel Internet of Things-Enabled Accident Detection and Reporting System for Smart City Environments

**DOI:** 10.3390/s19092071

**Published:** 2019-05-03

**Authors:** Fizzah Bhatti, Munam Ali Shah, Carsten Maple, Saif Ul Islam

**Affiliations:** 1 Department of Computer Science, COMSATS University Islamabad, Park Road Tarlai Kalan, Islamabad 44550, Pakistan; fizza687@gmail.com (F.B.); mshah@comsats.edu.pk (M.A.S.); 2 WMG, University of Warwick, Coventry CV4 7AL, UK; cm@warwick.ac.uk; 3 Department of Computer Science, Dr. A. Q. Khan Institute of Computer Science and Information Technology, Rawalpindi 47320, Pakistan

**Keywords:** Internet of Things, intelligent transportation systems, accident detection, smart cities

## Abstract

Internet of Things-enabled Intelligent Transportation Systems (ITS) are gaining significant attention in academic literature and industry, and are seen as a solution to enhancing road safety in smart cities. Due to the ever increasing number of vehicles, a significant rise in the number of road accidents has been observed. Vehicles embedded with a plethora of sensors enable us to not only monitor the current situation of the vehicle and its surroundings but also facilitates the detection of incidents. Significant research, for example, has been conducted on accident rescue, particularly on the use of Information and Communication Technologies (ICT) for efficient and prompt rescue operations. The majority of such works provide sophisticated solutions that focus on reducing response times. However, such solutions can be expensive and are not available in all types of vehicles. Given this, we present a novel Internet of Things-based accident detection and reporting system for a smart city environment. The proposed approach aims to take advantage of advanced specifications of smartphones to design and develop a low-cost solution for enhanced transportation systems that is deployable in legacy vehicles. In this context, a customized Android application is developed to gather information regarding speed, gravitational force, pressure, sound, and location. The speed is a factor that is used to help improve the identification of accidents. It arises because of clear differences in environmental conditions (e.g., noise, deceleration rate) that arise in low speed collisions, versus higher speed collisions). The information acquired is further processed to detect road incidents. Furthermore, a navigation system is also developed to report the incident to the nearest hospital. The proposed approach is validated through simulations and comparison with a real data set of road accidents acquired from Road Safety Open Repository, and shows promising results in terms of accuracy.

## 1. Introduction

It is recognised that cities are becoming increasingly crowded in terms of visitors, inhabitants and vehicles. The increase in the number of vehicles has led to an increase in traffic, which has led to an increase in the number of road traffic accidents. A recent World Health Organisation (WHO) report showed that every year 1.35 million people die and 50 million people get injured [1]. Road accidents are ranked as the eighth leading cause of deaths (up from ninth in its previous report in 2015), with the Association for Safe International Road Travel (ASIRT) predicting that it may rise to the fifth leading cause of deaths in the near future, unless drastic changes occur [2]. As well as the social harm caused by road traffic accidents, there is a significant cost. ASIRT estimates that between one and two percent of the annual budget of every country is spent on road accidents [2].

Recently, there has been a global increase in the annual number road traffic deaths, even in developed countries with good road safety measures [3]. However, it remains the case that the greatest burden of road traffic fatalities and injuries lies in low- and middle-income countries [4]. For example, in Pakistan, an average of 15 people lose their life each day to to such accidents. Information from the Pakistan Bureau of Statistics shows that 9582 accidents have resulted in 4036 deaths, demonstrating that there is, on average, more than one death in every two road accidents [5]. With such a rate of fatality, it is particularly important that road safety is improved in developed and, more importantly, developing countries. The emergence of the Internet of Things (IoT) gives promise for the development of intelligent traffic management systems.

Global Navigation Satellite Systems, such as the Global Positioning System (GPS), are being increasingly used in many applications, especially for vehicle positioning and navigation. Indeed, many vehicles that are shipped today have GPS devices that sense the position of the vehicle and send this information to cloud servers [6]. Other sensors, for use in accident detection or smart transport management, are also present in modern vehicles and continually acquire and store data [7]. High sampling rates, driven by a desire for increased accuracy and algorithm efficacy, lead to significant challenges in the storage and analysis of this data.

**Figure 1:**
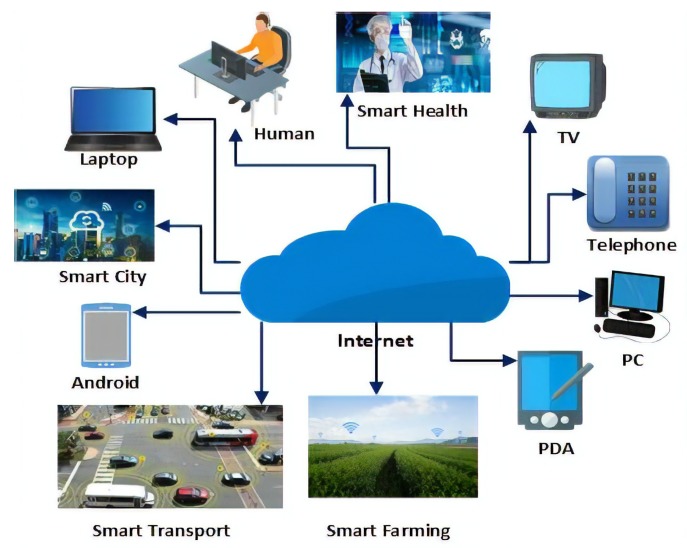
A generic IoT ecosystem comprising a variety of everyday objects.

**Figure 2:**
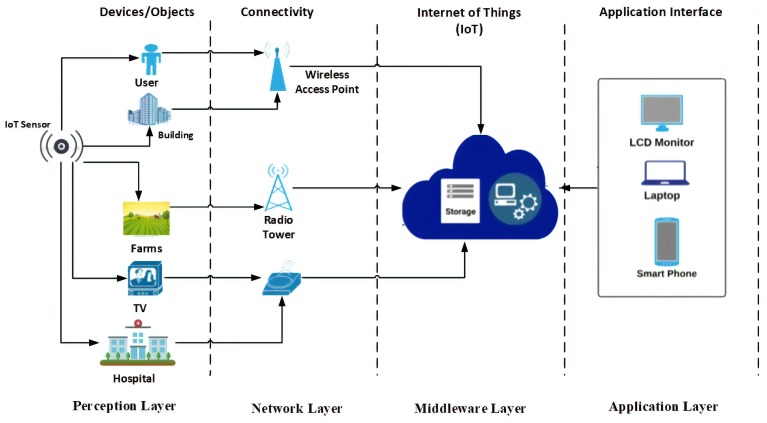
Basic IoT architecture.

There are many different definitions of IoT [8]. For example, in its Special Report on the IoT [9], the Institute of Electrical and Electronics Engineers (IEEE) describes the IoT as ‘a network of items—each embedded with sensors—which are connected to the Internet’. All definitions realise the IoT as a cyber-physical system that connects physical objects to cyberspace, such as in [10]. The extent and application of the IoT is significant and includes a variety of objects such as vehicles, buildings, mobiles, and different electronics appliances and infrastructural devices [11] or even clothes. Commonly, in an IoT system, a network connects devices—each with a unique identifier. These physical objects may have Radio-Frequency Identification (RFID) tags or other forms of identification, such as bar codes [12], and their presence is detected by a variety of sensors. These sensors take the object-specific information as an input and send it over the network to a system for processing and analysis. This processed data is then sent to decision-making units to determine automated actions to be invoked [13]. However, it should be recognised that sensors have limited computational power and storage capacity and this can create challenges, especially regarding security and trustworthiness. Cloud computing has been used to help overcome some of these issues [14]. Figure 1 presents an illustration of a generic IoT ecosystem and Figure 2 describes the basic IoT architecture.

The number and variety of advanced applications that utilize advanced technologies such as mobile computing, wireless communication, and sensing are vast and increasing. Examples include intelligent transport, cities, and disaster management systems, and many more that have become a hot research topic due to improved wireless communication technology. Ref. [15] decreased costs of storage and processing power, and availability and affordability of devices. Smart cities are being designed to provide better, more intelligent, reactive and cost-effective services to the population. Smart cities can provide mobility solutions through emerging intelligent transport systems [16]. Many countries are implementing real-time intelligent traffic systems to increase safety and reduce pollution. The economic and social benefits are clear; a recent World Bank study finds that “welfare benefits equivalent to 6 percent to 32 percent of the national GDP can be realized from reducing 50 percent of road deaths and injuries over a period of 24 years” [17]. In the field of intelligent transport systems, the main focus is on information obtained in real time and decision-making on the basis of this information [18].

One of the effective ways to reduce traffic fatalities is to reduce the response times to collisions. There are many systems, such as e-Notify, that can help in detecting and reporting traffic accidents [19,20]. The e-Notify system requires an onboard unit (OBU) in each vehicle. Whilst this may be an effective solution, it is expensive and not all vehicles are equipped with onboard units. The European Commission developed the eCall system and has decreed its mandatory deployment in every vehicle developed after 2015. The eCall system detects an accident and informs the emergency number 112 (999 in the UK and some other countries) [21].

The current solution that provides help in case of a vehicle accident is concerned with mostly one sensor. Amin et al. [22] propose a system that detects accidents automatically using GPS and notifies all the nearest hospitals and police. This is a hardware-based system and uses only one sensor to detect an accident; if this sensor fails, the whole system fails. Other systems use gravitational force to detect accidents and inform rescue teams. These systems, such as [23], have the same problem that they use only one sensor. Reliance on a single sensor also carries the risk of false positives—the reporting of an accident in the case that one did not occur. Other systems, such as [24], use accelerometer information as a trigger to notify emergency response about an accident.

Commercial organisations and the research community are both working to accurately detect accidents and provide timely assistance after an accident. The majority of systems being developed are hardware-based and this makes them expensive and not available in every car. There are a variety of situations in which the sensors in a vehicle may be damaged, including in minor collisions or stationary interactions. In such cases, the sensors are unable to detect an accident in this scenario. The reliability and availability of smartphone sensors make them an ideal candidate. Smartphone sensors can be used to detect accidents and are less likely to be damaged, resulting in false reporting of an accident. There are some existing systems that utilise smartphones to detect accidents. However, these systems have been found to have significant false positive rates. To overcome these issues, this paper proposes a novel IoT-based system that focuses on the accuracy of accident detection through low-cost devices. Our proposed system consists of two phases: accident detection and the notification system. This systems relies on the ubiquity of sensor-rich mobile phones for the detection of accidents. Our proposed system uses multiple smartphone sensors including accelerometer, GPS, pressure and microphone acquisition to detect accidents. We develop a smartphone application that continuously reads data from the sensors and transmits this information to the cloud for further computation. An accident is detected through threshold analysis. The main contribution of this paper is the development of a system that, upon detecting an accident, informs the nearest hospitals and ambulances. By using four sensory inputs, the system results in fewer false positives and more accurately detects accidents, outperforming previous methods.

The rest of paper is organized as follows: Section 2 presents the background on technologies such as IoT and Vehicular Ad Hoc Networks (VANETs) while Section 3 presents the state of the art. Section 4 proposes a model architecture for accident detection and reporting. In Section 5, the paper presents the proposed methodology where we elaborate on the working of the system. It is followed by Section 6 that explains the implementation of system. Finally, in Section 7, we present the experimental results of our proposed work, which is followed by conclusions and future work in Section 8.

## 2. Background

The IoT is an essential part of modern society. Increasingly, people want to stay connected with various things, and they have an increasing desire for automation. There are three fundamental forms of communication over the Internet: human-to-human; human-to-machine; and machine-to-machine. Until now, the majority of communication that takes place belongs to either human-to-human or human-to-machine communication types. The IoT is further enabling and enhancing machine-to-machine communication as a primary interaction type [25]. It is becoming an integral part of smart transport applications, smart cities, smart homes and smart industrial applications, see [26,27,28], and having significant impact on the academia, industry, government, and society [29].

**Figure 3:**
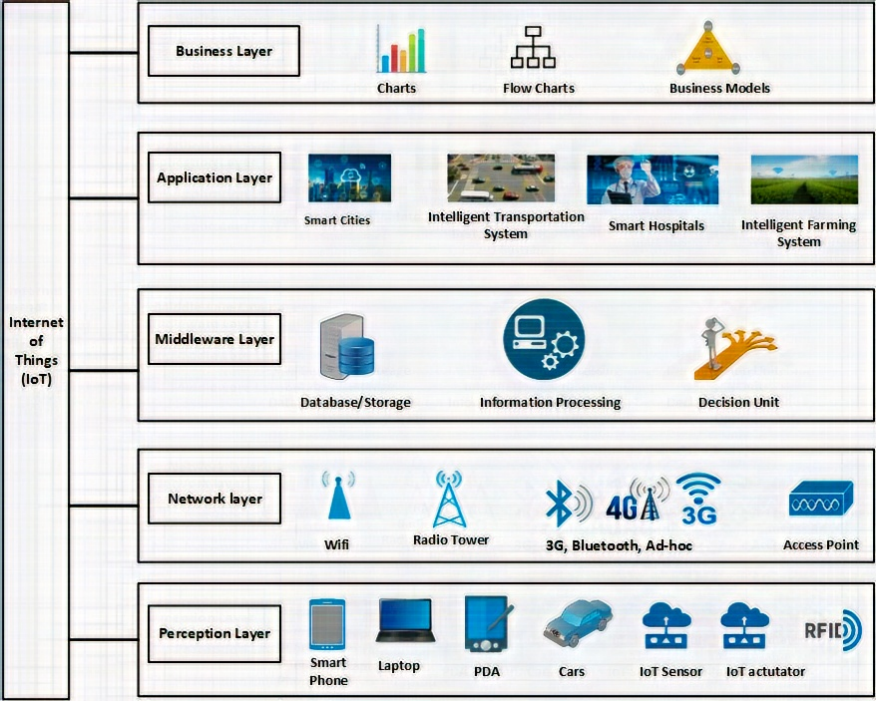
Generic architecture.

Different researchers have proposed different architectures for IoT systems. We favour the five-layer architecture concerning: the perception layer; the Network Layer; the Middleware layer; the Application Layer; and the Business or Logic Layer. These layers are described in [30,31]; these five layers as shown in Figure 3.

### 2.1. Perception Layer

This layer, also known as the “Device Layer” in other interpretations of IoT architectures, is responsible for interacting with everyday objects. The main purpose of this layer is to collect specific information related to objects and the environment. It may use RFID or Bar codes for object identification, and sensor technology or some other information collecting technique. This information may be related to location, temperature, acceleration, velocity, pressure, or chemical changes in the environment, for example. The type of information sent depends on the sensor type. This information is then passed to the network layer for further transmission [30].

### 2.2. Network Layer

This layer is basically a combination of “Network Access Layer” and “Network Transmission Layer”. It is also commonly known as “Transmission Layer”. The main purpose of this layer is to securely transfer information from the perception layer to the middleware later. The transmission methods used in the network layer include 3G/4G, Wi-Fi, Bluetooth and ZigBee, though many others such as Sigfox may be employed. The choice of transmission method also depends on the sensor device.

### 2.3. Middleware Layer

The main purpose of this layer is service management. The middleware layer helps to connect devices to provide a service. Each device communicates with devices providing the same service. This layer receives information from the Network layer and stores it in a database for any future use. This layer also performs the information processing in the processing unit, and can make decisions based on the information.

### 2.4. Application Layer

In this layer, applications are hosted which help in the manipulation of data aggregated in various ways. It provides the management of an object’s information collected from sensors. Different applications reside on this layer, such as building automation, continuous health-care monitoring, smart waste management, autonomous vehicles, to name a few.

### 2.5. Business Layer

This layer has the business models, organizational policies, and different types of authentication mechanisms in order to access and visualize the application layer data. These policies and visualizations are used for defining future actions and business strategies.

The demand for IoT systems and services is increasing. The population of cities is increasing steadily and this creates resource and organisational challenges as well as raising social issues and problems. According to the United Nation Population Fund, approximately 60% of the world population will live in cities by 2030 [32]. As a consequence of the increasing population, increasing traffic is creating a massive difficulty for citizens. Major budgets are allocated for construction of new roads and highways to relieve traffic congestion. Smarter transport systems, leveraging Internet of Things technologies, are seen as a fundamental part of the solution to such problems.

As the number of vehicles on roads increases, resulting in a greater number of collisions, so too does the importance of effective accident detection and response. A key aspect of a response strategy that requires addressing is the communication between a vehicle involved in an accident and emergency response units, once an accident has been detected.

Some researchers are paying attention to vehicle monitoring, with the purpose of addressing the issue around automated vehicle accident detection [24,33,34,35,36,37]. Different researchers have proposed different techniques for accident detection or accident alarm automation. Automatic detection through GPS is one of the key techniques that has been developed. The location of the vehicle is sent to “Map Matching Algorithm” and this algorithm detects the location of the vehicle on the road. It has been noted that such methods take a long time to notify hospitals or emergency services [22]. Hu et al. [38] use a technique based on 3D models to predict accidents. The sample data includes motion trajectories which are collected through the 3D model.

Neural algorithms essentially learn the motions of a vehicle and then match this with sample data. It predicts an accident by matching the trajectories with learned activities. Chandran et al. [39] have proposed a helmet that can detect and report an accident using sensors and cloud computing infrastructure. Sensors send the data to the emergency contact numbers. Other research has proposed a decision support system for the routing of the vehicles without including accident detection or notification features [40]. Research has also been proposed in the area of IoT-based accident detection techniques. This method detects the accident and informs all the WhatsApp numbers in the driver’s mobile phone [41].

The major problem with this method is that it is a very time-consuming process. The application first informs the personal contact number and then the person informs the hospital to send an ambulance or safety team [41]. Nasr et al. [42] propose a method that informs a central headquarters about the accident. This method has the same issue that there is a time delay, and this method is applied to only a single vehicle.

In this paper, a smart vehicle accident rescue technique has been developed wherein an accident is detected through the sensors of an Android smartphone. Sensors send this data to an IoT architecture that processes that data and informs the nearest hospitals about the accident. The system also guides the nearest ambulance or safety service in reaching the site of the accident. This technique can be applied to any type of vehicle. The system is capable of sharing data between the cloud and emergency service providers in real time. This data is shared in order to enhance the rescue aspects of the system. The system is highly accurate and as such there are very few false positives.

## 3. Literature Review

There are many schemes and techniques in the literature to address road safety, vehicular communication and rescue operations after an accident. We focus on the most practical solutions and restrict attention to the techniques that are software- and hardware-based systems. We primarily focus on the methods for accident detection that use multiple sensory inputs. This section presents an analysis of existing systems related to traffic hazards and road accidents, highlighting their strengths, weaknesses and limitations. The main features and drawbacks of each technique or method are presented in Table 1.

### 3.1. Smart Phone-Based Systems

In the literature, we can find a significant amount of research attempting to address the problem of low-cost retrofitted solutions for identifying and notifying of vehicle accidents, based on mobile phone technology. Zhao et al. [43] have proposed a crash notification systems that utilises mobile devices, detecting accidents through accelerometer and GPS data. This system delays in sending a message about an accident. Reddy et al. [44] proposes the technique that detects the accident, using the position of the vehicle and informs of an accident by sending an SMS from the mobile. This system uses only one sensor, namely the position of the vehicle, and this can lead to false reporting of accidents. In [45], the authors propose a system that uses the gravitational force, speed, and noise to detect an accident. An emergency notification is sent to a web server that then sends an SMS to the emergency contact number. This system is the closest to our proposed system, using some of the same sensors. The main weakness of this system is that there is a possibility of false reporting of an accident at low speeds, where the system struggles to ascertain reliably whether the user is in the vehicle.

Patel et al. [46] developed an Android application that detects accidents using only accelerometer data. The system automatically sends a pre-recorded voice message to the 108 ambulance service (an emergency service available in India, like 112 or 999 in other countries). Aloul et al. [47] also focus on the accelerometer as the main sensor in a smartphone for the detection of an accident. This system continuously receives data from the accelerometer and use this to determine the severity of an accident. It notifies the the medical service provider of accident location and sends information about the owner/driver. The problem with both of these systems is their reliance on a single sensor gives a tendency for false reporting since there is no other information to corroborate a suspected accident. Khot et al. [48] propose a smartphone based system that detects an accident using an accelerometer and finds the nearest emergency point to send the location of the accident. Again, this system has the problem of a single point of failure leading to a tendency of false reporting.

Zaldivar et al. [34] have developed a smartphone application using an on-board unit. This application enables the driver to speak with his/her vehicle. The application detects an accident using air bag triggers and informs the emergency service provider through email or SMS. A drawback of this application is that it requires the smartphone application to be running. Faiz et al. [49] also proposed a vehicle accident detection and an alarm system using a smartphone. This system detects an accident using the pressure sensor in the phone. They measure the speed using GPS and the tilt of angle using the accelerometer on a smartphone. This systems detects the accident using the two sensors: GPS and accelerometer through the smart phone. The data regarding the accident is stored on the server. The system is more reliable than some others, but failure can arise in the case of server failure [50]. When the system identifies an accident has taken place, it informs the nearest hospital and police station. Thompson et al. [35] propose a system that detects accidents using the sensors in a smartphone. The phone uses its 3G connection to send the accident information. This system is not fully automated as it sometimes requires a third party to send the emergency information to the responders.

Rajkiran et al. [24] propose a new technique that automatically detects the collision using the accelerometer and informs the emergency dispatch server along with relevant information using Global System for Mobile (GSM) messaging. Again, this system only uses a single sensor to detect the accident. Namrata et al. [36] propose a vehicle accident detection and tracking system utilizing GPS and GSM. PUSH ON SWITCHES sense the accident and send the location to the user-defined phone number using GSM.

Prabha et al. [37] proposed another method that provides an automatic localization system using GPS. The method also provides a communication service using a GSM modem. The system detects an accident using the accelerometer, which triggers a message to be sent to police headquarters and to a rescue team. If a small accident occurs, the driver can terminate the alert message. Dogru et al. [51] propose a system that uses a smartphone to continuously share data related to the position and speed of the vehicle to other vehicles. Different machine learning algorithms are used to analyse the data and provide information regarding road conditions and for accident detection. This system tries to make decisions on the basis of the data that has been gathered. Unfortunately, the results show poor accuracy in accident detection in the paper.

A further research work that is of interest was created by PoP et al. [52]. The authors developed a smartphone-based application that uses the built-in accelerometer and gyroscope for the detection of accidental falls and informs the nearest emergency provider for first aid assistance. The proposed system focuses on reducing the response time and does not, however, consider accidents in vehicles.

### 3.2. Hardware-Based Systems

Traffic accidents are, as stated, a major cause of fatalities and so a great deal of research is underway to automatically and immediately identify accidents and for rapid initiation of rescue operations. If the time between accident and dispatch of the rescue team is reduced there is a lower likelihood of fatality and this has led many researchers to work on reducing the response time. Young [53] has presented an analysis of cell-phone usage and accidents through analysing OnStar data. The OnStar system involves an embedded mobile telephony card that calls for assistance upon driver activation or automatically through an airbag deployment. Clearly, this system has the limitation that it requires manual intervention, or is only activated in times of serious incident where the airbag is deployed—and, of course, relies on the sensor that indicates that the airbag should be deployed.

Some systems operate in certain locations or use cases. Intersections are a common place for collisions since there are several conflicting movements from a different direction. Ki et al. [54] propose a system that automatically detects, records and reports accidents at intersections. Cameras are placed on the intersections to detect the vehicle and its related information, such as velocity, speed, area, and direction. The decision taken by the system is on the basis of the features extracted. We can discover the reasons for an accident, as well as features of intersections that impact safety. Obviously, intersections are the only place where this system works and it will not detect accidents at other non-intersection locations. In Tushara et al. [55], the authors propose a system for accident detection that utilises a micro-controller to control all operations. Messages are sent to a predefined mobile number. The performance evaluation of this system demonstrated false reporting of accidents. The system present is for accident detection only and does not concern any rescue system.

Fogue et al. [56] propose the intelligent system that is able to detect the accident and notify the emergency services. This system also estimates the severity of an accident to reduce the assistance time by using data mining and knowledge inference techniques. The severity is used in decision-making for emergency rescue resource utilization. Challenges that are faced in the implementation of the system include latency, bandwidth and delivery guarantees. In the paper, Chaturvedi et al. [57] propose an accident detection and reporting system that identifies accidents with the help of one sensor. When an accident is detected, a message is sent to the nearest police station and relatives of the victim. Liang et al. [58] propose a system that uses support vector machines (SVM) and IoT to efficiently detect accidents. This system is for accident detection and also traffic prediction.

Maleki et al. [59] propose a GPS-based location tracking system. This system collects accident information through the use of a crash sensor. It sends information through SMS messaging to an emergency centre. The emergency centre then dispatches a response team to the accident location. Despite all its advantages, the system has some drawbacks too. For instance, the system is not fully automated and the user has to start the system manually. The IoT-based system proposed by Nasr et al. [42] detects accidents using a shock sensor. This system provides basic information regarding accidents to the rescue team. This system helps the rescue team to recognise an accurate location of the accident. The system also facilitates determination and communication of the shortest and best route, which is then sent to the nearest ambulance. This is not scalable and is limited to a small number of accidents. Another limitation of the system is that, due to its reliance on a single sensor, accuracy of detection is very low.

Table 1 describes the main features and limitations of various techniques. This table gives the details of the evaluation parameter used in each paper. The tools and implementation method are also presented in the table.

As we can clearly see from the limitations summarised in Table 1, many systems lack accuracy in accident detection. Moreover, many systems use an onboard processing unit, increasing the cost of the overall system and making it less feasible for widescale adoption. Using smartphone sensors can decrease the overall cost of a system and makes it more accessible to users.

A variety of smartphone-based solutions have been proposed by researchers.

Table 2 presents details of the sensory inputs to the accident detection and response systems. As can be seen, the maximum number of sensor types used in current systems is 3, and only one system using this many types of input.

In summary, many researchers have proposed techniques or systems for automatic accident detection, reporting, and rescue. The majority of the systems are dependent on smartphones, though there are a number of proprietary products that have been developed. These latter systems usually involved a manual activation aspect and direct calls to call centres only. Our propose a system that utilises five sensors and is composed of following components: Accident Detection; Communication; Rescuing; and Navigation. Our system focuses on the accuracy of accident detection.

## 4. Proposed Architecture

**Figure 4:**
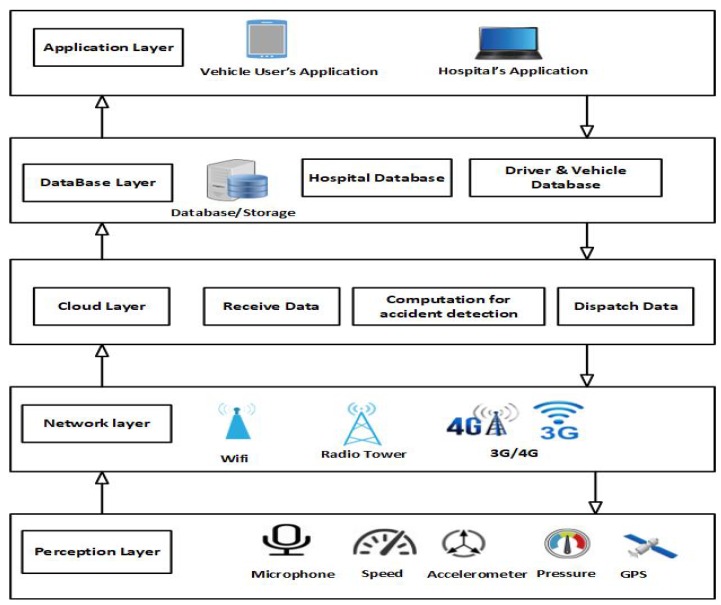
Architecture of ADRS.

In order to address the current limitations in accident detection systems, we propose a novel Accident Detection and Reporting System (ADRS). ADRS uses the capabilities of a modern Android smartphone thereby decreasing the overall cost since there are no special hardware requirements. All processing is performed in the cloud. The architecture of the ADRS is a layered architecture as is seen in Figure 4.

The system architecture of ADRS is comprised of five different layers, namely the Application layer, the Database layer, the Cloud layer, the Network layer, and the Perception layer. In the proposed architecture, the perception layer is responsible for interacting with the sensors of the smartphone. The main purpose of perception layer in the ADRS architecture is to collect sensor data from the sensors. This information is related to gravitational force, speed, sound, pressure, and location of the vehicle. All is derived from the sensors in the smartphone. These data are then passed to the network layer for further processing. The network layer is responsible for providing the connectivity between the perception and cloud layers. This layer receives data from the smartphone sensors, location, and driver information from the sensors available in the perception layer. The network layer utilises WiFi or 3G/4G cellular communications for the transmission of data to the cloud layer. The cloud layer holds the algorithm for accident detection and identifies an accident on the basis of threshold analysis. If an accident is identified, it informs the nearest hospital about an accident. The processing layer transfers data to the database layer. Finally, the database layer is responsible for storing the data related to the accident , hospital information, driver, and vehicle information. All of the information is transferred to the application layer, which includes the smartphone application interface for the driver and interface of the web-based system for the hospital.

**Figure 5:**
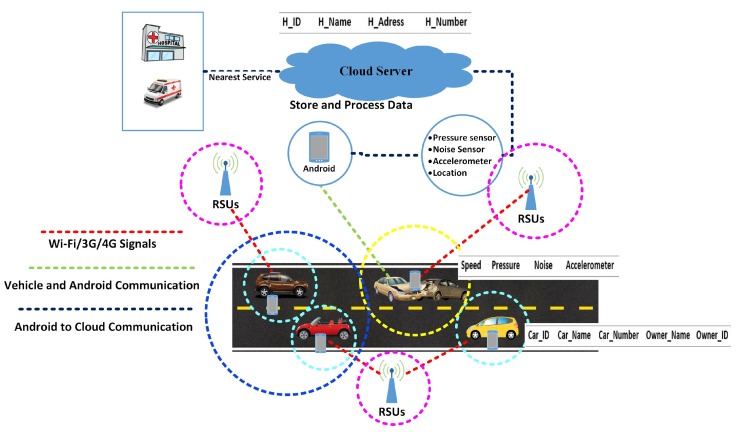
Working of ADRS.

Figure 5 shows the working of the proposed system to give a better understanding of system. A user (vehicle driver) downloads the application from the Google Play Store and installs it on their smartphone. After installation, the user registers the application, providing the required information; after registration, the user can use the application freely. Each time the user commences a journey, he or she activates the tracking process. The smartphone begins reading the sensor data and sends this to the cloud. This information is then processed in the cloud with the aim of detecting any accidents. In the case of an accident, a nearby hospital is informed and presented with details about the accident.

The system assumes that each car is connected to the smartphone. Each smartphone is equipped with four types of sensors: a pressure sensor; a noise sensor (microphone), an accelerometer, and a speed sensor. For the experimental evaluation, we have used an Android phone equipped with the aforementioned sensors which continuously collect raw information from these sensors. The phone continually sends the data to the cloud which processes the data and looks for an accident condition.

In the proposed system, roads are furnished with roadside units (RSU). These RSUs are used to cache the information from cars. In our test scenario, we have five vehicle names as V1, V2, V3, V4, and V5, respectively. Vehicle V1 communicates with the nearest RSU; without loss of generality, we can name this RSU_1. In our scenario, vehicle V2 also communicates with RSU_1 because it is not within the range of RSU_2 and RSU_3. Vehicle V3 and V4 collide, resulting in RSU_1 being within range. The information of the accident cannot directly be shared with the RSU_3 because it is out of range, as is the case with vehicle V2.

**Figure 6:**
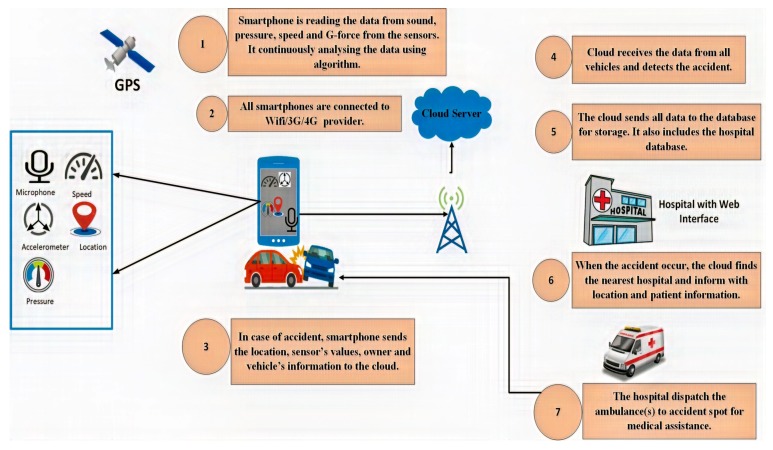
Overview of the proposed system.

The cloud processes data and checks whether an accident has occurred or not. Threshold values are defined and, if the values collected from sensors give rise to a value that exceeds the threshold value, it indicates that an accident has occurred. When these conditions are reached, an alarm is generated and sent to the vehicle driver. If the driver cancels the alarm, then the hospital is not informed, in order to avoid false reporting. If the driver does not respond within 10 s, the cloud service sends a notification to the nearest hospital. The cloud contains a database of cars and hospitals. The hospital sends an ambulance to the accident location for rescue operation. The hospitals also hold a database with information on the ambulances. The main objective of this architecture is to enhance the accuracy of accident detection. This system consists of two phases: (I) the accident detection phase; (II) the notification phase. These phases will be further discussed in the following sections. Figure 6 shows the overview of the system.

**Figure 7:**
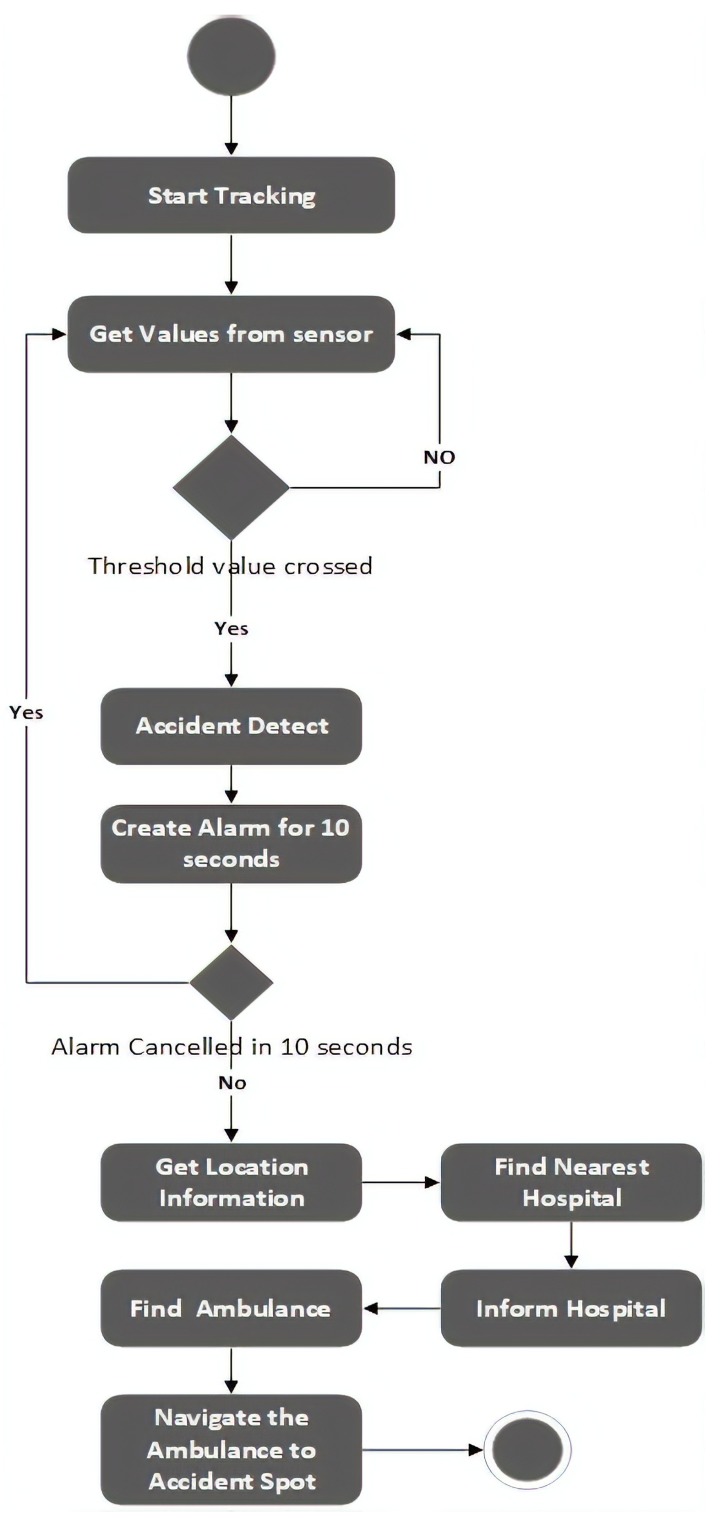
Flow diagram of the proposed system.

The fundamental goal of our system is to provide an architecture that: (1) allows direct Vehicle to Infrastructure(V2I) communication; (2) automatic exchange of information regarding the accident; (3) to enhance the accuracy of accident detection; (4) to reduce the number of false reports; and (5) develop a cost-effective system. Figure 7 shows the activities involved in proposed architecture.

### 4.1. Phase 1: Accident Detection

#### Accident Detection Components

**Figure 8:**
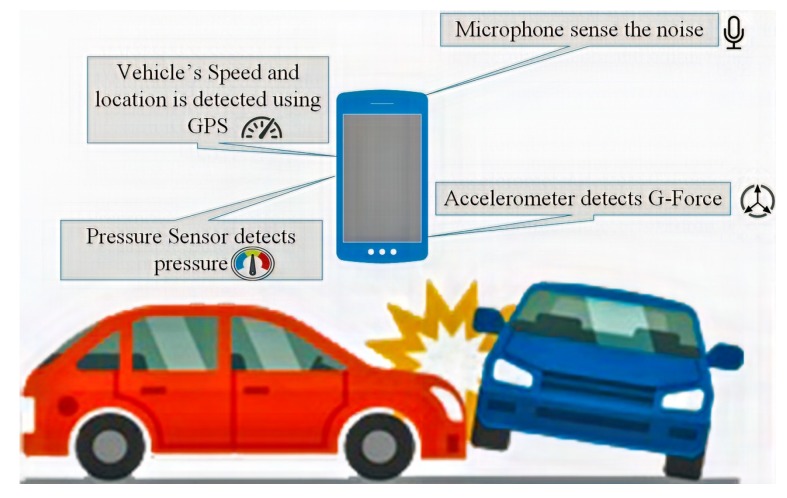
Components for accident detection.

Accident detection is used to prevent unfortunate incidents that result in damage or injury and hence reduce death rates from road traffic accidents. Figure 8 illustrates the basic components used in the accident detection phase. These components help in identifying the occurrence of an accident. The detection phase utilises the data from the smartphone: microphone, accelerometer, GPS, and pressure sensor to determine the occurrence of an accident. The following are further details on the components used in the accident detection phase.

- **Smartphone Accelerometer sensor:** This component is used to detect accelerometer sensor information to find out the acceleration force (or G-force). The accelerometer in a smartphone is one of the essential components to detect an accident. If the acceleration force is greater than 4 G, an accident flag is raised [35]. It should be noted that, to detect an accident accurately, the G-force data is insufficient in isolation. Furthermore, the threshold value of 4 G is derived through a combination of secondary research and experimentation. When the phone is dropped inside or outside of the vehicle, readings of 2 G or 3 G are observed [11]. If a vehicle stops suddenly but is not in an accident (say under emergency braking), it encounters under 1 G of force. By considering all cases, 4 G is determined as a threshold to raise an accident flag. This threshold value helps to avoid any false positives [62].
- **GPS Technology:** This component is used to extract the Global Navigation Satellite System (GNSS) positional data through the GPS system. The GPS system identifies and tracks the position of the vehicle and forwards that data through the system. The GPS data can also be used as a determinant of speed and can be used to determine the speed of the vehicle. The probability of accurately identifying an accident is increased by considering the speed of the vehicle, since at different speeds the factors such as noise generated and deceleration experienced are different to higher speed accidents. If the speed of the vehicle is less than 24 km/h, there would not be dramatic changes in the speed unless there was an accident. Previous experiments have shown that the variance in the distribution of the speed value is greater than 2.06 in times of an accident [45,62]. To identify an accident, we therefore consider that, when the speed is less than 24 km/h and the variance is greater than 2.06, we need to check the noise and gravitational force. If the vehicle is moving at low speed and the noise and gravitational force are sufficient, then an accident is identified.
- **Pressure sensor:** A pressure sensor is used to detect the pressure of a car in collision. This component also collects the continuous data of pressure and it raises an accident flag when the pressure exceeds a prescribed threshold of 350 Pa. The pressure sensor is used to enhance the accuracy of the system and to reduce the chances of false identification and reporting of an accident.
- **Smartphone microphone:** This component is used to detect the ambient sound. An accident flag is raised when the noise exceeds the threshold value, which is 140 dB. Since we are using accelerometer and pressure sensor to help identify an accident with increased accuracy. The built-in microphone is used to improve the accuracy and to reduce the probability of false positives. The built-in microphone is used to sense sound. At the point when a vehicle collides, a built-in microphone could sense high decibel sounds. However, it should be recognised that there is a possibility that noise is simply made by the passengers laughing, the mobile dropping or loud music. In line with [35,45,62], the sound threshold value is 140 dB. The microphone is used to increase the probability of accurate detection of an accident.

### 4.2. Phase 2: The Notification Phase

**Figure 9:**
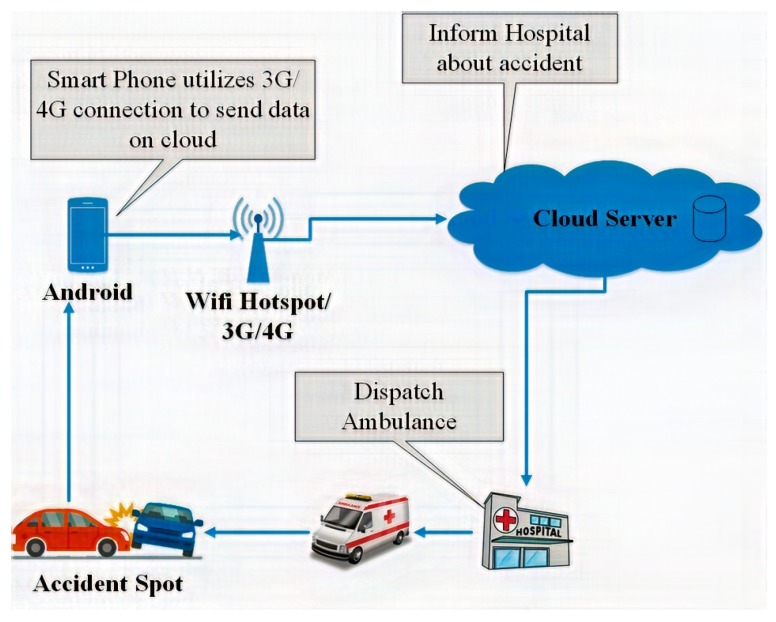
Components of the notification system.

Whilst detection of accidents is a significant and important problem, effective notification and dispatch is also important; relevant accident information must be dispatched immediately to the emergency rescue team. When an accident is identified, the system gets the location of the accident from the GPS of the smartphone. The 3G/4G data connection of the smartphone is utilized to send accident information, such as G-Force value, pressure value, noise value, speed value, and location to the cloud. The cloud has a database of hospitals and determine the nearest hospital using a mapping service (in our case, the Google Maps API). A notification is sent to the hospital with details of the location, along with owner information. The data collected is stored into the existing database. Figure 9 explains the operation of the notification phase.

### 4.3. Databases

1. **Car Database:** A car database contains all necessary information about the cars that have been registered. For example: owner information (Computerized National Identity Card (CNIC), Name, Address) and a car number is stored in the cloud to address any mishap (see Table 3).
2. **Hospital Database:** To inform the hospitals of an emergency, the system needs to know all nearby hospitals. When the system sends a message to the cloud, the cloud needs to find the nearest hospital and forward the message to it. The information stored is provided in Table 4.

## 5. Proposed Methodology

In this section, we explain the prototype of the proposed system. A complete system is developed that comprises two main components: the smartphone Android application and the web-based system. A smartphone application is built using Android Studio (3.3.1, Google & JetBrains, Mountain View, CA, United States). We use the Android application to access the smartphone accelerometer and microphone to provide data on the G-force and ambient noise, and to gain a pressure sensor value. An accident is identified based upon these values.

1. **Connection** The user starts the application on the Android phone, having enabled Wi-Fi/3G/4G. The application commences the collection of data from three sensors, accelerometer, microphone, and pressure sensor of the smartphone as well as recording GPS data.
2. **Accident Detection** The proposed detection phase can be stated by the following equation [45], where AD is the accident detection pointer flag. In the equation:
  (a) **AC** is an acceleration value that is detected by the smartphone.
  (b) **Noise** is the value of noise which is detected by the microphone of the smartphone.
  (c) **SVP** is a speed variation period that is used to detect accidents at low speed.
  (d) **Accident Threshold** (AT) is an accident detection threshold. This is set to 1.5.
  (e) **Speed** (S) is the event speed value, calculated using the G-Force.
  (f) **Low-speed threshold** (LST) is a value to detect accidents at low speed, set to be 3.
  (g) **MP** is a maximum period of time for consideration of low speed accidents:
    (1) $$AD=\{\begin{array}{cc} 1, & \mathrm{if}\;\left(\left(\frac{AC}{4G}+\frac{\mathrm{Noise}}{140}+\frac{\mathrm{Pressure}}{350}\right)\right)\geq \mathrm{AT}\wedge \left(\mathrm{Speed}⩾24-km/h\right),\\ 1, & \mathrm{if}\;\left(\left(\frac{AC}{4G}+\frac{\mathrm{Noise}}{140}+\frac{\mathrm{Pressure}}{350}+\frac{SVP}{2.06}\right)\right)\geq \mathrm{LST},\\ 1, & \mathrm{if}\;\left(\left(\frac{AC}{4G}+\frac{\mathrm{Noise}}{140}+\frac{\mathrm{Pressure}}{350}\right)\right)\geq \mathrm{AT}\wedge \left(\mathrm{Elapsed}\;\mathrm{Time}<MP\right),\\ 0, & \mathrm{otherwise}. \end{array}$$
  The cloud processes the data using the formula above to detect an accident. When an accident is identified, an alarm is raised. The user can press the cancel button within 10 s in the case this is a false flag, in order to avoid a false report of an accident. In the case that the message is not cancelled, an emergency alert message is sent to the nearest hospital. The algorithm of accident detection is described in Algorithm 1.
3. **Notification** After the confirmation of an accident, the smartphone finds the geographical location of the accident using the smartphone GPS receiver. We use the Google Maps API to discover the location of the collision. The system determines the nearest hospital and utilizes the Wi-Fi/3G/4 G cellular data connection to send collision information such as vehicle number and the location of the accident to the nearest hospital for rapid recovery. The algorithm of accident detection is described in Algorithm 2.

**Algorithm 1:** Algorithms for accident detection.

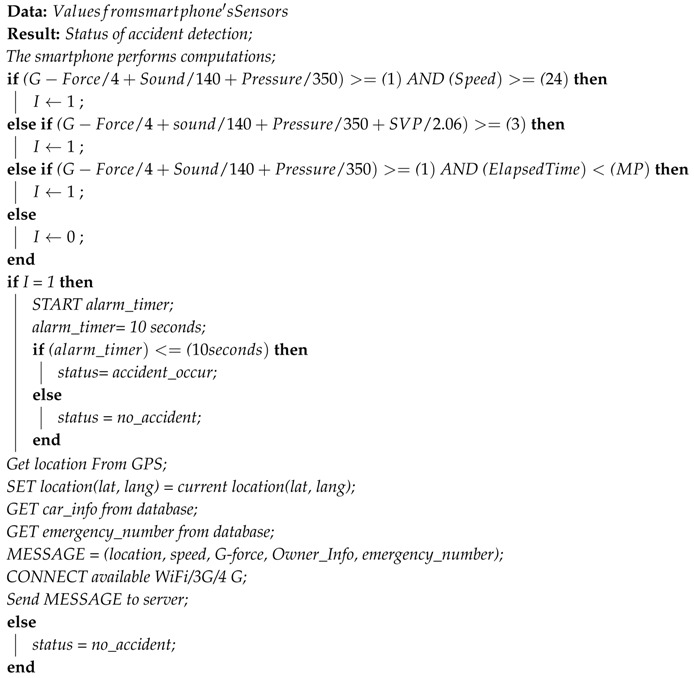



**Algorithm 2:** Algorithms for notification **Data**: MESSAGE=(location,Owner_Info,Vehicle_Info) **Result**: *Ambulance Dispatched;* *Server decode the message;* *lat1 = start.lat;* *lon1 = start.lng;* *lat2 = end.lat;* *on2 = end.lng;* *dLat = lat2 - lat1;* *dLon = lon2 - lon1;* a= Math.sin(dLat / 2) * Math.sin(dLat / 2); b= Math.cos(this.toRad(lat1)) * Math.cos(this.toRad(lat2)); c= Math.sin(dLon / 2) * Math.sin(dLon / 2); d= a+b*c; e= 2 * Math.atan2(Math.sqrt(d), Math.sqrt(1 - d)); Dist= R * c; *Cloud finds the nearest hospital using the*
***HAVERSINE**;* *Hospital= nearest_hospital;* *Update the data base;* *Server sends notification to Hospital Web Interface;* *Hospital dispatch the ambulance;*

## 6. System Implementation

As explained, our system comprises two phases: accident detection and notification phase. For the accident detection phase, a smartphone application has been fully implemented. For the notification phase, a web-based system has been implemented for use by hospitals.

### 6.1. Detection Phase Implementation

**Figure 10:**
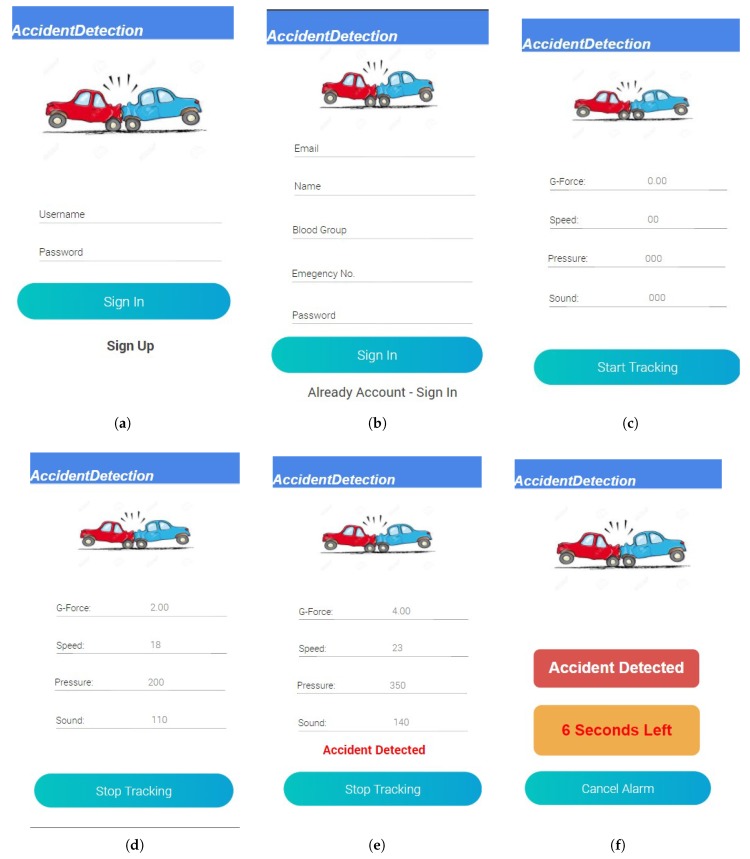
Android Application. (**a**) Sign In Screen; (**b**) Sign Up Screen; (**c**) Start Tracking; (**d**) No Accident; (**e**) Accident Detected; (**f**) Alarm.

An Android application has been developed in the Java programming language, using Android Studio version 3.3.1. The application is developed for an Android operating system with minimum API level 17 and target API level 26. A user first registers for system use. Once registered, to use the system, the user enters their ID and password to log in to the system. Recording and transmission of data commence when the user clicks to start tracking. The application continually reads the data from the smartphone’s sensors and sends the data to the cloud. If an accident is identified, the application generates an alarm for 10 s. Figure 10 shows the interfaces of smartphone android application. The smartphone application consists of the following activities:

1. Start and Stop Accident Detection Activity,
2. Tracking of Accident,
3. Cancellation of Alarm,
4. Management of Account.

### 6.2. Notification Phase Implementation

**Figure 11:**
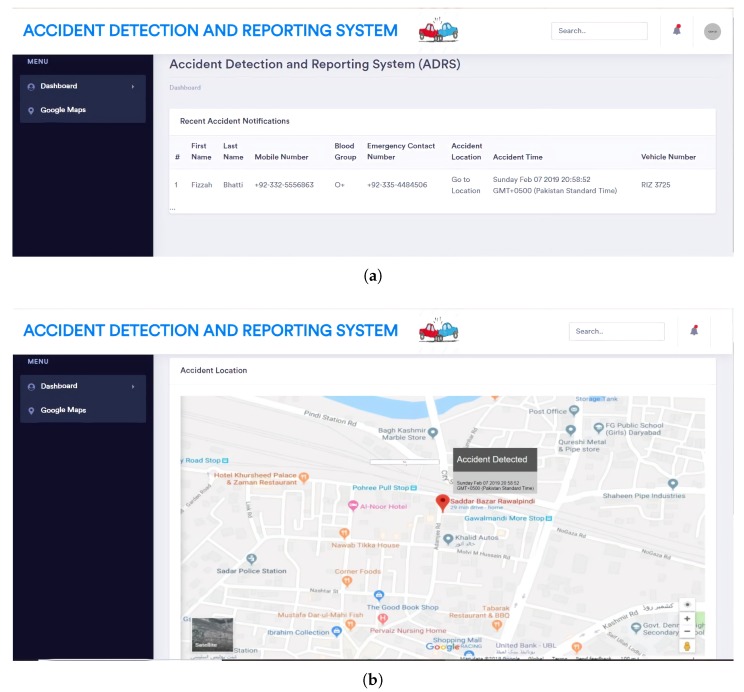
Experimental results. (**a**) accident details; (**b**) location of the accident.

After an accident is identified, the cloud determines the nearest hospital and informs the hospital about the accident. This is performed using a web-based application. The application has been developed using ASP .NET MVC 4. This interface is used by the hospital to establish whether there is an emergency or not. Whenever an accident occurs, the website receives the information regarding the accident. The website shows the details of accident such as the location of accident and driver and vehicle information. A Microsoft SQL database is used to store all the information regarding an accident. The website uses HTML, CSS and bootstrap for the development of the interfaces. The Google Maps API is used to show the position of the accident on a map. Figure 11 shows the working of web based application.

## 7. Experimental Results

Clearly, the accident-detection algorithm cannot be tested in a real, live environment due to safety and damage issues. Indeed, even testing in controlled environments is challenging and costly. However, we have conducted extensive testing through simulation in a controlled environment. The system records acceleration on three axes—speed value, noise value and pressure value at the highest possible rates. This data is sent to the cloud and the cloud processes this data to enable accident detection. The simulation code and data can be found at Supplementary Materials.

### 7.1. Threshold Evaluation

It is stated that it is not possible to detect the accident in a real environment. To evaluate the threshold, we conducted the experiment in a real environment. We used real vehicles and drivers, driving at different speeds. We simulated the other factors such as speed, noise, G-force, and pressure. We aimed to imitate the real environment in the vehicle as is required to test the Android application.

**Figure 12:**
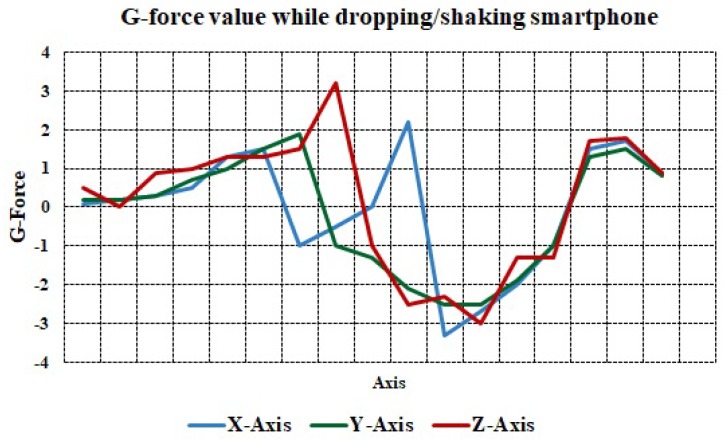
G-force value while dropping a smartphone.

The smartphone would experience a G-force of more than 4 G in the case of an accident. To mimic this situation, we dropped the smartphone forcefully inside the vehicle to reach the threshold of 4 G. In our experiments, we could not achieve an acceleration of 4 G. Figure 12 shows that, by dropping the smartphone in the vehicle, the maximum force experienced is not more than 3.3 G.

### 7.2. Comparison with OnStar System

The OnStar [63] system is selected as a comparison to our proposed ADRS. The OnStar system has been created by General Motors (GM) (Detroit, MI, United States) for on-road assistance. The system uses the on-vehicle sensors that activates whenever the accident occurs and informs the emergency provider. OnStar is selected because it is a hardware-based system that uses in-vehicle sensors. The system is only used by GM vehicles and cannot be installed in all brands of vehicles. Table 5 shows the comparison of both systems.

### 7.3. Comparison with CADANS

In this section, we present the results of our experiments when evaluating the accuracy of our smartphone application. A C# based simulator is created to evaluate the accuracy. Our performance is compared to the car accident detection and notification system (CADANS) [45], that detects accidents on the basis of smartphone sensors: accelerometer, GPS, and microphone. We simulated the behaviours of CADANS using three sensors and our proposed solution (ADRS) with four sensors. The results show that ADRS outperforms CADANS in terms of accuracy. There are some cases where CADANS does not detect identify an accident but an accident has occurred. The simulator we have developed, an Accident Detection Simulator (ADSim), simulates a mobile ad hoc network and uses threads to manage multiple vehicles. All sensors have their own get and set classes, and generate values every other second. The simulation runs for two minutes and, after that, results are analyzed. Experimental setup details are given in Table 6. Values are generated for each sensor and formulas for accident detection are executed. The simulator indicates for which values actual accidents occur.

**Figure 13:**
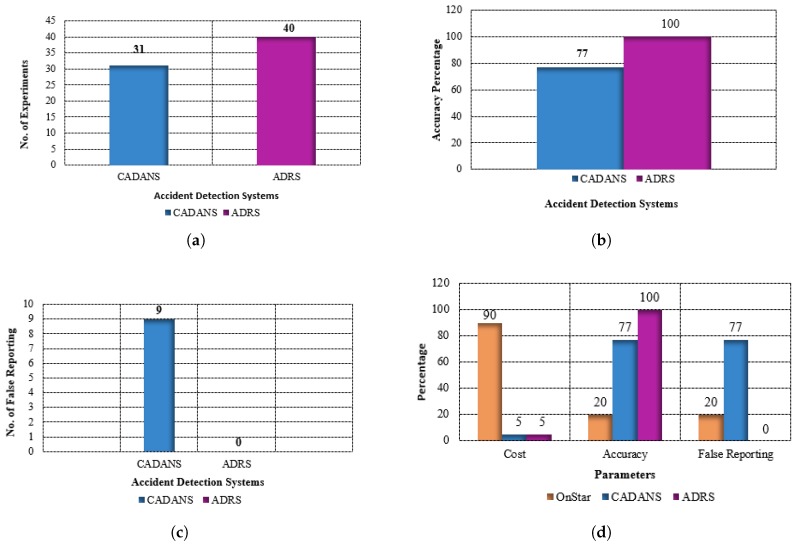
Experiment results. (**a**) comparison of accident detected; (**b**) accuracy percentage of experiments; (**c**) false reporting of experiments; (**d**) parameter based comparison.

The simulation is conducted for two minutes. In this period, 40 accidents occur and ADRS detects all 40 accidents, whereas CADANS accurately detects 31 accidents. CADANS also generates more false reports than ADRS. CADANS detects an accident in some cases where the G-force is lower than 3 G (which as explained, may be due to a phone being dropped). CADANS reports nine incidents as accidents that are not actual accidents. The added use of the pressure sensor data in ADRS decreases the probability of false reporting. Figure 13 shows the results of simulations.

### 7.4. Evaluation with the FODR Dataset

We have also conducted experiments on a dataset for accident detection acquired from the Find Open Data Repository www.data.gov.uk. This is a dataset of accidents that have occurred in 2016. Speed and noise values were extracted from the dataset. The aim of using this data is to collect the speed and noise values arising in actual accidents. Table 7 shows the actual values and accident occurrence.

We now consider the performance of accident detection systems employing a variety of sensors. There are three cases that we consider:

**Case 1:** In this case, we compare the extracted real speed value with the system that has only one sensor [56]. This system cannot detect the accident which occurs at a speed less than 24 km/h.

**Case 2:** In this case, we compare the situation where we have two sensors [45]. We consider the speed and noise sensor. This system does not identify an accident at a lower speed in this case in which an accident did actually occur. When the speed is greater than 24 km/h, it may identify an incident as an accident, where in reality there was no accident. If a vehicle is at a low speed, but the noise exceeds the threshold, the system may not consider it as an accident, where, in reality, there was an accident.

**Case 3:** In this case, we use multiple sensors; we use the accelerometer, speed, noise and pressure sensors to identify an accident. By using multiple sensors, we increase the accuracy of accident detection; there is a reduced chance of both a false negative and false positive. It is also possible to detect the accidents at low speeds which are missed in other systems. The following table shows the results of our proposed system.

Table 8 shows the comparison of accident detection using the three systems. Case 1 can detect three of the five incidents. In case 2, where the noise sensor is also used detects four out of five accidents, failing in the accident that is at low speed. In case 3, we used a combination of speed, accelerometer, noise, and a pressure sensor and all accidents are identified, even the one at low speed.

**Figure 14:**
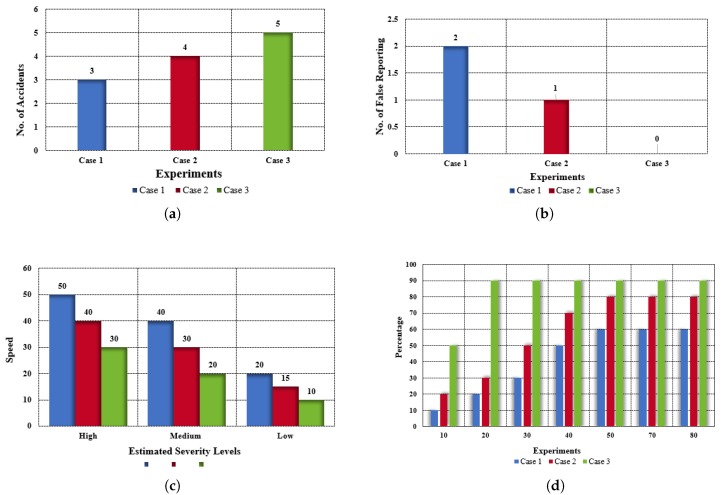
Experimental results. (**a**) Comparison of experiments for the three cases. (**b**) False reporting in the three cases. (**c**) Estimated severity of accident for the three cases. (**d**) Accuracy percentage in the three cases.

In further tests, Case 3 (the proposed system), 90% of accidents are detected accurately. Figure 14d shows the accuracy. Figure 14a shows the comparison of the three cases. Figure 14c shows the severity of the accident at different speeds. From the data, we can observe that the severity of the accident is high in the 30–50 km/h speed range; the severity of the accident is medium from 20–40 km/h and very low below 20. We performed five experiments and in terms of false reporting, Case 1 gives two false reports, Case 2 gives one, and Case 3 gives zero false reports (Figure 14b).

## 8. Conclusions and Future Work

In modern cities, the volume of vehicles has increased drastically in recent years. This increased traffic has resulted in an increase in the number of accidents. While there exist a number of accident detection systems being brought to market, still a significant number of fatalities arise. At least part of this problem is due to the lack of a timely response to serious accidents, caused by inadequate automatic accident detection and inefficient notification and routing of emergency response. The lack of availability of effective systems, for affordability and retrofitting capability issues, only exacerbates the problem. To address these issues, we propose an IoT-based system for accident detection. We have shown that using a variety of different sensors can help in detecting a road accident more accurately. The proposed system immediately detects the location of an accident and calculates the nearest hospital and sends an emergency request for assistance to the required hospital department. This system takes the decision on the basis of data received from smartphone sensors, detecting information about the vehicle status. We have demonstrated that our proposed approach reduces the number of false alarms seen in earlier work. Our system requires Internet connectivity to function. The limitations of our study include that we have conducted the primary evaluation of the system in a simulated environment. In the near future, we will enhance the system by introducing mobile edge computing to reduce latency and enhance security and privacy. Indeed, the system requires a full security and privacy analysis, and we intend to address this in future work.

## Figures and Tables

**Table 1:** Summary of literature review findings.

Ref.	Features	Limitations	Evaluation Parameter	Tools
[43]	Detects accident using accelerometer and GPS	Single point of failure	Accuracy	Actual Implementation
[44]	Accident detection based on position of vehicle	Single point of failure	Response Time	Actual Implementation
[45]	Accident detection and reporting system	No resource estimation	Accuracy	Actual Implementation
[46]	Detects accident using accelerometer	Single point of failure	Accuracy	Actual Implementation
[47]	Use Accelerometer for detection	Single point of failure	Response Time	Actual Implementation
[52]	Use accelerometer & Gyroscope for detection	Single point of failure	Response Time	Actual Implementation
[48]	Finds the nearest emergency point	Single point of failure	Response Time	Actual Implementation
[34]	Accident detection and rescue system	Manual system	Efficiency	Real vehicle
[49]	Accident detection using a smartphone	Single point of failure	Accuracy	Actual Implementation
[50]	Detects accident using two sensors	Single point of failure	Accuracy	Actual Implementation
[35]	Accident detection using mobile phone	Involvement of the third party	Response Time	Google ION device
[24]	Accident detection via accelerometer	Single point of failure	Response Time	Actual Implementation
[36]	Accident detection and alarm system	Single point of failure	Response Time	Simulation
[60]	Path planning and controlling the traffic lights	No guarantee of smooth travel	Accuracy	Empirical Result
[37]	Detects the accident via accelerometer	Single point of failure	Accuracy	GSM and GPS modem
[51]	Detects the accident via speed	Single point of failure	Response Time	GSM and GPS modem
[54]	Detects accidents at the intersection	Only valid on intersections	Accuracy	Actual Implementation
[55]	Informs about the collision	Informs only one mobile number	Response Time	Actual Implementation
[53]	Accident detection via air bag	Inform only to the emergency number	Accuracy	Actual Vehicle
[22]	Detects the accident using the GPS speed	False reporting of accident	Response Time	GSM and GPS modem
[56]	Detects severity of the accident	Delay in the message sending	Accuracy	Prototype
[57]	Detects accident and reporting system	Based on one sensor	Accuracy	Aurdino Implementation
[58]	Detect accident via vector machine	Not a rescue system	Efficiency	Real World Traffic Data
[59]	Detects the accident using crash sensor	Congestion issue on the server	Response Time	Actual Implementation
[42]	Detects the accident and the shortest path	Single point of failure	Reliability	Simulations
[61]	Detects & Report Accident	False Reporting	Response Time	Testbed

**Table 2:** Summary of sensor types used in existing systems.

Ref.	Accelerometer	Speed	Pressure	Sound	GPS	Other	Total
[43]	✔	✕	✕	✕	✔	✕	2
[44]	✕	✕	✕	✕	✔	✕	1
[45]	✔	✔	✕	✔	✕	✕	3
[46]	✔	✕	✕	✕	✕	✕	1
[47]	✔	✕	✕	✕	✕	✕	1
[52]	✕	✕	✕	✕	✕	✔	1
[48]	✔	✕	✕	✕	✔	✕	2
[34]	✔	✕	✕	✕	✕	✔	2
[49]	✕	✕	✔	✕	✕	✕	1
[50]	✔	✕	✕	✕	✔	✕	2
[35]	✔	✕	✕	✕	✔	✕	2
[24]	✕	✕	✕	✕	✔	✔	2
[36]	✕	✕	✕	✕	✔	✕	1
[60]	✕	✕	✕	✕	✔	✕	1
[37]	✔	✕	✕	✕	✕	✕	1
[51]	✕	✕	✕	✕	✕	✕	0
[54]	✕	✕	✕	✕	✕	✔	1
[55]	✕	✕	✕	✕	✕	✔	1
[53]	✕	✕	✕	✕	✕	✔	1
[22]	✕	✕	✕	✕	✔	✕	1
[56]	✕	✔	✕	✕	✕	✕	1
[57]	✔	✕	✕	✕	✕	✕	1
[58]	✕	✕	✕	✕	✕	✕	0
[59]	✕	✕	✕	✕	✕	✔	1
[42]	✕	✕	✕	✕	✕	✔	1
[61]	✔	✕	✕	✕	✕	✔	2
**ADRS**	✔	✔	✔	✔	✔	✕	5

**Table 3:** A car database.

Car_ID	Car_Name	Car_Number	Owner_Name	Owner_ID
C1	Suzuki Mehran	RIZ 3725	Bilal Khalid	34512-4520645-5
C2	Mazda	MN 3909	Usman Bhatti	32103-9963008-2
C2	Toyotta Carolla	LEL 06 4520	Ali Haider	12345-1529307-7

**Table 4:** A hospital database.

H_ID	H_Name	H_Address	H_Number
H1	Jinnah Hospital	Usmani Rd Faisal Town Lahore Punjab	+92-42-99231443
H2	Ali Medical Center	Kohistan Road F8-Markaz Islamabad	+92-51-2255313
H3	Military Hospital	Abid Majeed Rd Rawalpindi Punjab	+92-51-9270346

**Table 5:** Comparison of OnStar and ADRS.

Parameter	OnStar [63]	ADRS
Automatic Detection	✔	✔
Probability of False Positive	High	Less
Range	Only for GM vehicles	For each vehicle
Applicability	USA	Whole World
Cost	$59.99/month	Free
Pre-Hardware deployment	Required	Not Required

**Table 6:** Details of ADSim.

Parameter	G-Force	Speed	Sound	Pressure
Ranges	1–10	20–30	130–150	300–400
At start	0.00	0.00	0.00	0.00
Thresholds	4.00 G	22–24 km/h	140 dB	350 P

**Table 7:** Base value of accident detection.

Experiment No.	Speed Value	Noise Value	Accident Detection
1	20	130	✔
2	20	135.5	✔
3	30	170	✔
4	40	184.5	✔
5	50	200	✔

**Table 8:** Comparison of systems using different numbers of sensors for detecting accidents.

Experiment No.	Speed	Actual Detection	Case 1	Case 2	Case 3
1	20	✔	✕	✕	✔
2	20	✔	✕	✔	✔
3	30	✔	✔	✔	✔
4	40	✔	✔	✔	✔
5	50	✔	✔	✔	✔

## Supplementary Materials

The simulation code and data are available online at https://github.com/FizzahBhatti12/ADRS_Data.

## Abbreviations

The following abbreviations are used in this manuscript:

ITS	Intelligent Traffic System
IoT	Internet of Things
DSS	Decision Support System
GPS	Global Positioning System
RFID	Radio-Frequency Identification
GSM	Global System for Mobile
km/h	kilometer per hour
SVP	Speed Variation Period
dB	Decibels
ADRPS	Accident Detection and the Reporting System
RSU	Road Side Unit
AC	Acceleration
AT	Accident Threshold
LST	Low Speed Threshold
MP	Maximum Period of Time
V	Vehicle
VANETs	Vehicular Ad Hoc Networks
GM	General Motors
IEEE	Institute of Electrical and Electronics Engineers
GNSS	Global Navigation Satellite System
CNIC	Computerized National Identity Card
